# Supplementary material for: Regulatory and evolutionary impact of DNA methylation in two songbird species and their naturally occurring F1 hybrids
Source: BMC Biol. 2024 May 29;22:124. doi: 10.1186/s12915-024-01920-2 (PMC11134931; doi:10.1186/s12915-024-01920-2)
Supplement: Supplementary file 2 — Additional file 2: Supplementary Results. Fig. S1. Figure 1A-L in main manuscript colored by sample group (species and hybrids). Fig. S2. Methylation gene profile with gene body split into exons and introns. Fig. S3. Tissue-specific patterns of the association between genetic- and methylation differentiation. Fig. S4. CGI proportion gene profile. Fig. S5. Patterns of genetic and epigenetic change at misexpressed genes. Table S2. Frequency of hypermethylation of tissue-specific DMRs. Table S7. Classification system for the mechanism of DNA methylation divergence. Table S8. Number of fixed difference loci by divergence class. Table S9. Number of differentially expressed genes. Table S10. Determinants in cis of misexpressed genes. [file 12915_2024_1920_MOESM2_ESM.pdf]

Regulatory and evolutionary impact of DNA methylation in two songbird species and their naturally occurring F_1 hybrids

Jesper Boman, Anna Qvarnström, Carina F. Mugal

Supplementary Information

1. Supplementary Results
2. Figure S1 – Figure 1 A-L in main manuscript coloured by sample group (species and hybrids)
3. Figure S2 – Methylation gene profile with gene body split into exons and introns
4. Figure S3 – Tissue-specific patterns of the association between genetic- and methylation differentiation
5. Figure S4 – CGI proportion gene profile
6. Figure S5 – Patterns of genetic and epigenetic change at misexpressed genes
7. Table S1 – sample and sequencing information (see external file)
8. Table S2 – Frequency of hypermethylation of tissue-specific DMRs
9. Table S3-6 – tsDMR GO analyses (see external files)
10. Table S7 – Classification system for the mechanism of DNA methylation divergence
11. Table S8 – Number of fixed difference loci by divergence class
12. Table S9 – Number of differentially expressed genes
13. Table S10 – Determinants in *cis* of misexpressed genes

Supplementary Results

1. Gene annotation

To improve annotation of untranslated regions (UTRs) and better predict transcription start sites (TSS) and transcription termination sites (TTS), we updated the gene annotation of the collared reference genome FicAlb1.5. For this purpose, we used 36 RNA-seq samples from six tissues (brain, heart, kidney, liver, testis and spleen) of the six collared flycatcher individuals for the multi-assembly Oyster-River protocol [1] for *de novo* transcriptome assembly as well as MAKER [2]. In total, 14,943 out of 16,576 genes mapped to the collared flycatcher chromosome-level assembly. Of these, 9,597 genes had at least one 5' UTR (in the following referred to as promoter set) and 8563 also had a 3' UTR (gene profile set).

2. Relationship between tsDMRs and tissue-specific expression

To understand the impact of tsDMRs on gene expression levels we quantified tissue-specific gene expression using the preferential expression measure (PEM; Figure SR1) [3]. We tested for a significant deviation from random rank of PEM for genes with a tsDMR in a certain tissue. If tsDMRs are associated with tissue-specific expression, then we expect to see an excess of genes with tsDMRs in the promoter having either the highest or lowest expression. Only tsDMRs at CGI promoters in testis (COL, PIE and HYB) and brain (HYB) had a significant deviation from random PEM ranks (χ^2 test of independence, $p < 0.05$), which means that there is an association between tsDMRs and tissue-specific expression. For these, we tested for a significant difference in PEM between genes with a tsDMR in a certain tissue and genes with no tsDMR in any tissue (reference set). This test answers the question: do genes with a detectable tsDMR in the promoter show greater tissue-specific expression than genes lacking promoter tsDMRs? This forms a test of the relative importance of DNA methylation compared to other unobserved explanatory variables for tissue-specific expression patterns. Testis, which had the highest number of tsDMRs overlapping CGI promoters, showed a significantly higher PEM in overexpressed genes compared to the reference set (Wilcoxon test, $p < 0.05$). This highlights the importance of DNA methylation in conferring testis-specific expression, since the reference set consisted both of genes with ubiquitous expression and genes whose tissue-specificity is controlled by mechanisms other than tissue-specific promoter methylation. Underexpressed genes with tsDMRs did not show lower PEM values, indicating that tsDMRs in promoters are generally associated with tissue-specific overexpression in this tissue.

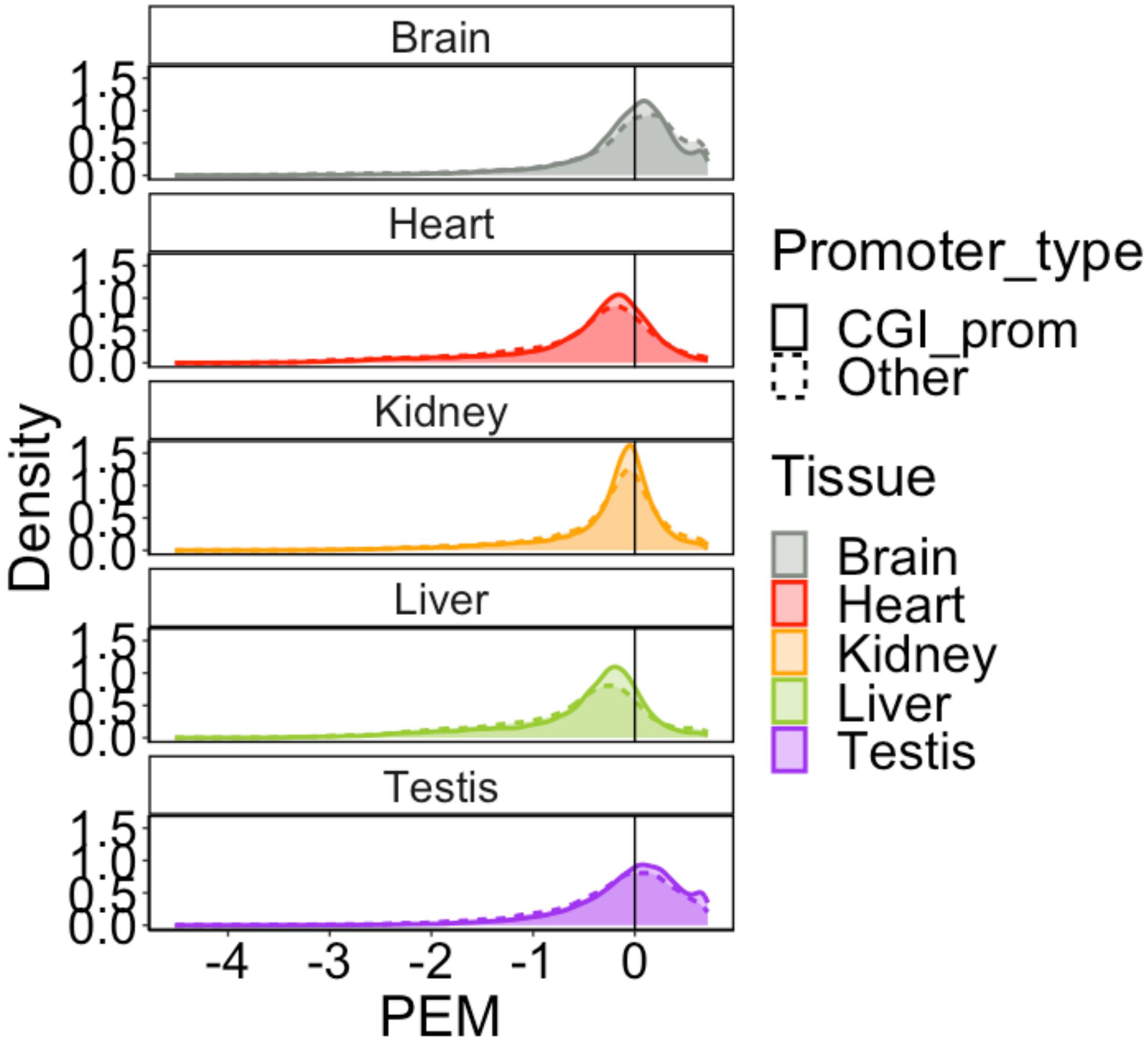

Figure SR1. Distribution of tissue-specific expression values per promoter type and tissue. A PEM value >0 and <0 indicates overexpression and underexpression compared to other tissues respectively.

3. Tissue-specific hybrid inheritance patterns of promoter DNA methylation

We investigated promoter methylation further because of its role in transcriptional repression. When comparing samples from the parental species using PCA, tissue was a more important factor to divergence in promoter DNA methylation compared to species (Figure SR2A-B). When we split up the dataset by tissue, the PCA separates the species with hybrids grouping intermediately along the first and second PC axes (Figure SR2C-L). This intermediate placement is distinct from earlier observations based on expression data, where misexpression in the hybrids dominated the PCA in all tissues except testis (Mugal et al. 2020).

To further understand the effects of DNA methylation divergence in promoters of hybrids we compared their methylation level with the parental species (see Supplementary Methods). This allowed us to classify the inheritance pattern of DNA methylation into six classes: conserved (HYB close to both COL and PIE), collared-dominant (HYB closer to COL), pied-dominant (HYB closer to PIE), and the two mismethylation categories, overdominant (HYB higher than

COL and PIE) and underdominant (HYB lower than COL and PIE). For CGI promoters 81-95 % were classified as conserved per tissue compared to 48-76% for Other promoters (Fisher's exact test, $p < 0.05$, Figure SR2M). In contrast, the relative proportion of non-conserved inheritance classes were similar between promoter types and only significantly different for heart ($p \approx 0.033$) and liver ($p \approx 0.013$; Figure SR2M). For both heart and liver, more overdominance in *Other* promoters was driving the significance. Around 1-4 % of CGI promoters were mismethylated per tissue compared to 7-11 % for *Other* promoters, further highlighting the stronger conservation of CGI promoters (Figure SR2M). Distribution of inheritance patterns were significantly different between tissues both with (Fisher's exact test, $p < 0.05$) and without brain, which was sequenced to lower coverage (Table S1). Liver, for example, showed the highest excess of Collared-dominance while heart and testis had excesses of over- and underdominance respectively. These results were in line with genome-wide results indicating that hybrid inheritance of DNA methylation varies predictably among tissues (Table 1). Overall, the intermediate placement of hybrids in-between parental species in the PCA (Figure SR2), is explained by a mixture of collared- and pied- dominant as well as additive effects (Figure SR2M).

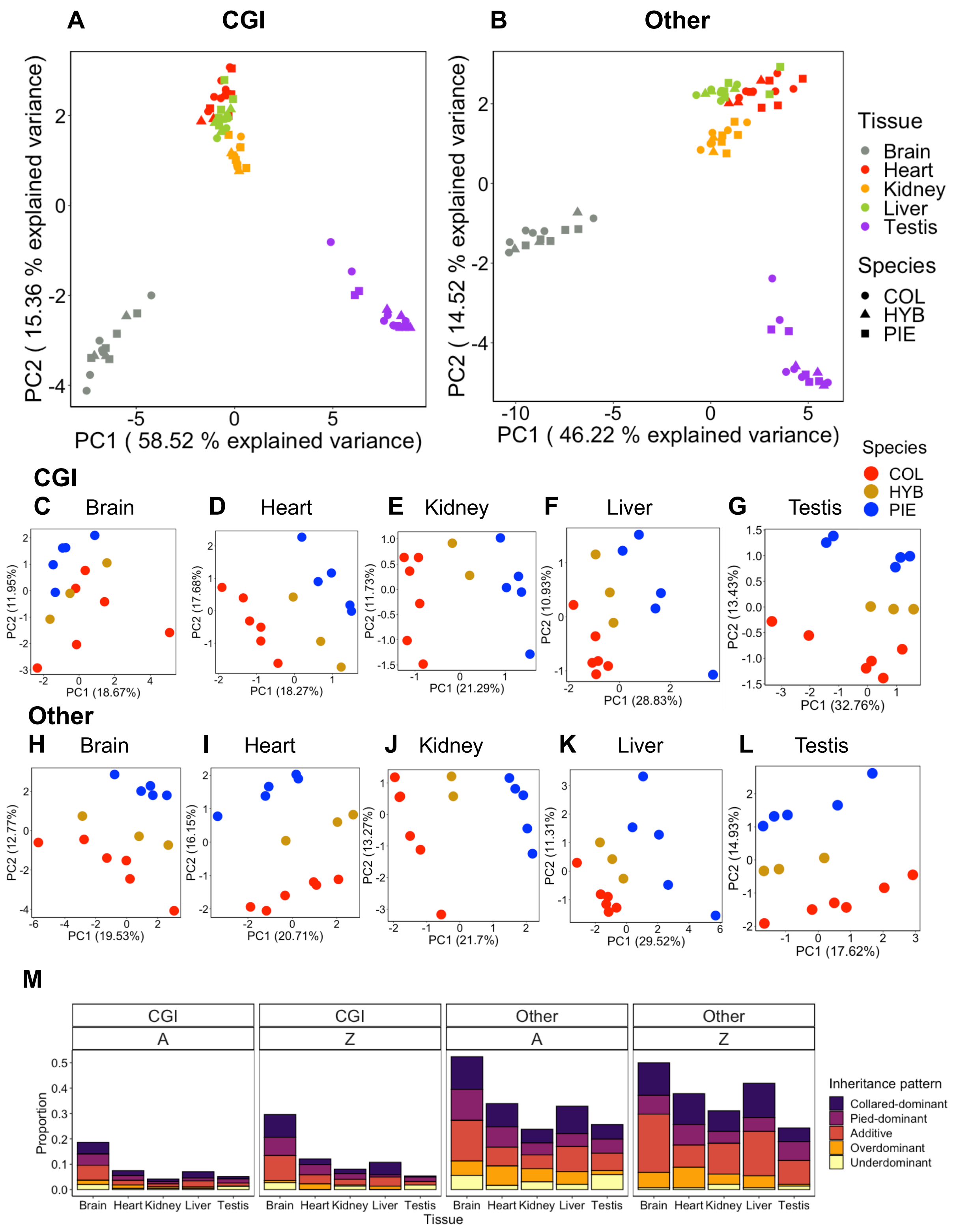

Figure SR2. Principal component analysis of promoter methylation patterns and inheritance patterns of methylation in F1 hybrids. CGI promoter methylation PCA for all samples (A) and separated by tissue (C-G). *Other* promoter methylation PCA for all samples (B) and separated by tissue (H-L). When including all samples, tissue dominates methylation variation regardless of promoter type. When separating per tissue, hybrids generally show intermediate promoter methylation levels. The inheritance pattern of promoter methylation shows that this

intermediate placement is mainly due to a combination of additive, Collared-dominant and Pied-dominant effects (M).

Previous work on the *Ficedula* flycatchers has shown a greater genetic differentiation on the Z sex chromosome compared to the autosomes (A) [4], as well as divergence in gene expression [5]. While all tissues and promoter type combinations showed a tendency towards less conservation on the Z chromosome, none of these differences were significantly different (Fisher's exact test, $p > 0.05$). Instead, a slightly higher proportion of additive effects on the Z sex chromosome compared to autosomes where significant for brain (CGI) as well as kidney and liver (*Other*) (Figure SR2M). This may be due to slightly greater methylation differentiation on the Z chromosome (<2 percentage points for all tissues; Wilcoxon rank sum test, $p < 0.05$; Figure SR3), to some extent perhaps caused by greater genetic differentiation (*t*-test; $p < 2.2 * 10^{-16}$) on the Z compared to autosomes (Figure SR4). There was also significantly more collared-dominance on Z compared to A in liver and brain (CGI), more pied-dominance on Z for heart (CGI), and less underdominance on Z in brain and testis (*Other*).

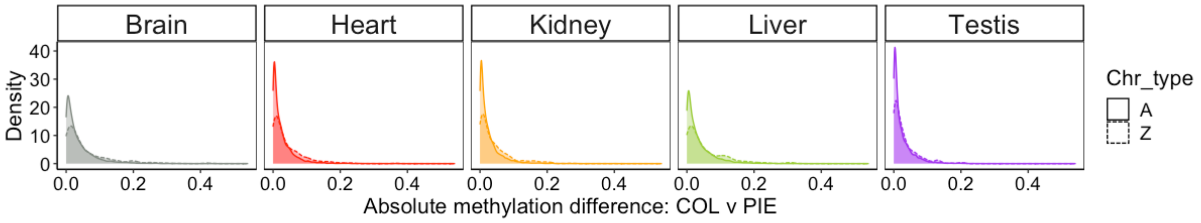

Figure SR3. Methylation differentiation (M_{diff}) of promoter sequences on autosomes (A) and the Z chromosome.

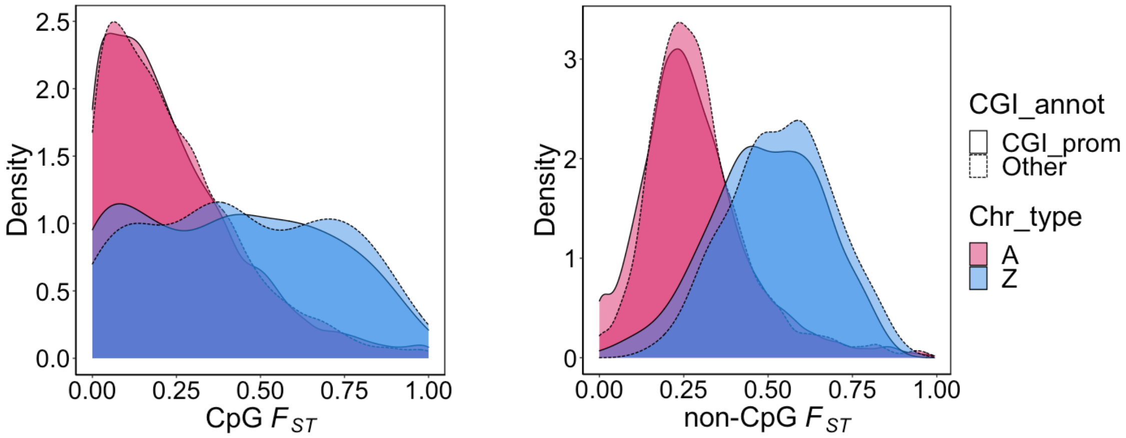

Figure SR4. Genetic differentiation (F_{ST}) in the promoter is higher on the Z compared to the autosomes.

Supplementary Information References

1. MacManes MD. The Oyster River Protocol: A multi-assembler and kmer approach for de novo transcriptome assembly. PeerJ. 2018;2018:e5428.
2. Holt C, Yandell M. MAKER2: An annotation pipeline and genome-database management tool for second-generation genome projects. BMC Bioinformatics. 2011.

https://doi.org/10.1186/1471-2105-12-491.
3. Huminiecki L, Lloyd AT, Wolfe KH. Congruence of tissue expression profiles from gene expression Atlas, SAGEmap and TissueInfo databases. BMC Genomics. 2003;4:1–10.
4. Ellegren H, Smeds L, Burri R, Olason PI, Backström N, Kawakami T, et al. The genomic landscape of species divergence in Ficedula flycatchers. Nature. 2012;491:756–60.
5. Mugal CF, Wang M, Backström N, Wheatcroft D, Ålund M, Sémon M, et al. Tissue-specific patterns of regulatory changes underlying gene expression differences among Ficedula flycatchers and their naturally occurring F1 hybrids. Genome Res. 2020;31:1727–39.

Figure S1: Figure 1 A-L of the main manuscript but colored by sample group (species and hybrids). The results show that COL, PIE and HYB show similar patterns.

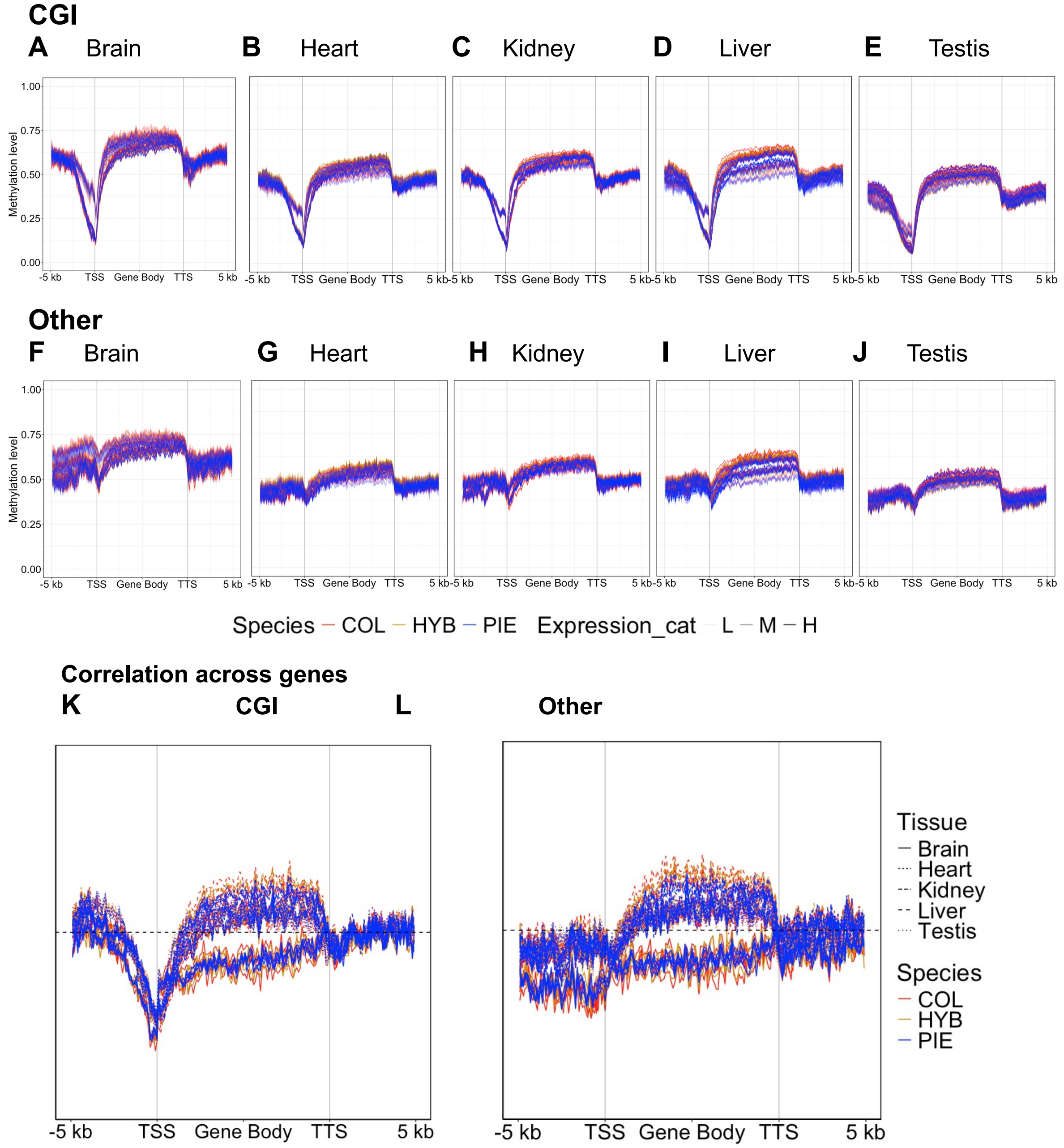

Figure S2: Methylation level at CGI (A-E) and *Other* (F-J) promoter types across five tissues with gene body split into intronic and exonic sequence. In general, the methylation level is higher in exons than introns. Orange lines represent intronic sequence and red lines represent exonic sequence. Solid lines represent COL, short-dashed lines HYB and wide-dashed PIE.

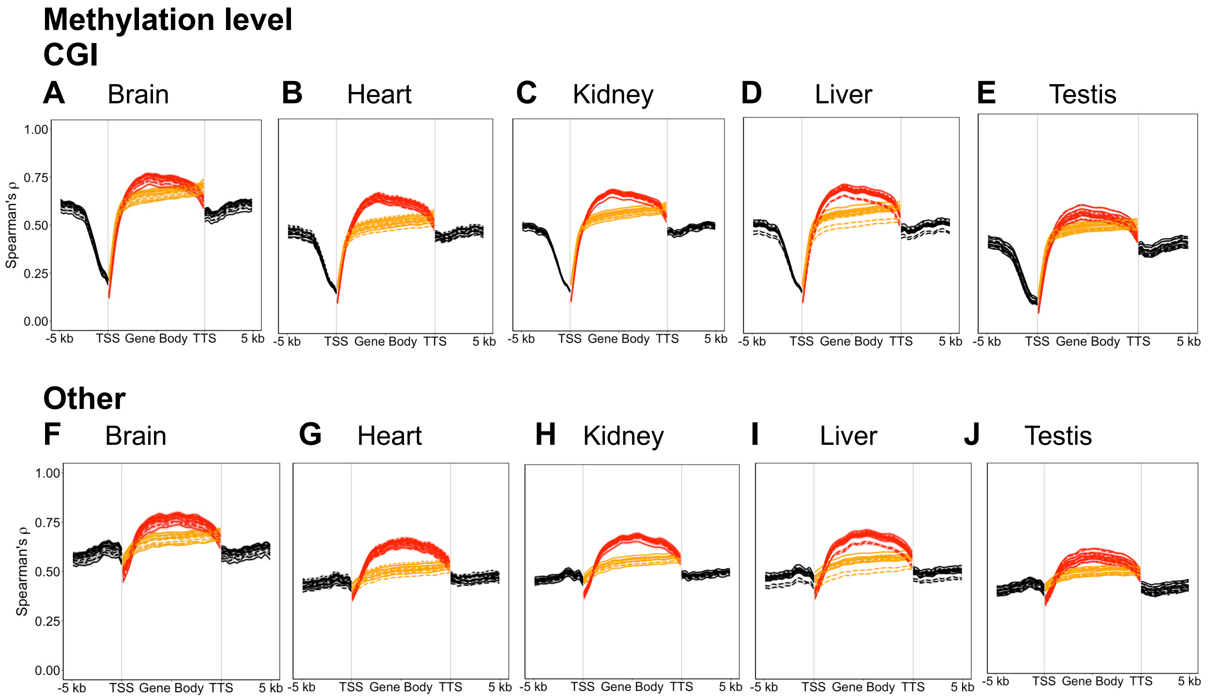

Figure S3: Tissue-specific patterns of the association between genetic- and methylation differentiation. (A) and (B) are the same panels as Figure 3A and D of the main text respectively but colored by tissue instead. The results show that tissue both has an impact on the relationship between M_{diff} and F_{ST} and that impact is not explained by neither F_{ST} (which is the same regardless of tissue) nor the average level of M_{diff}.

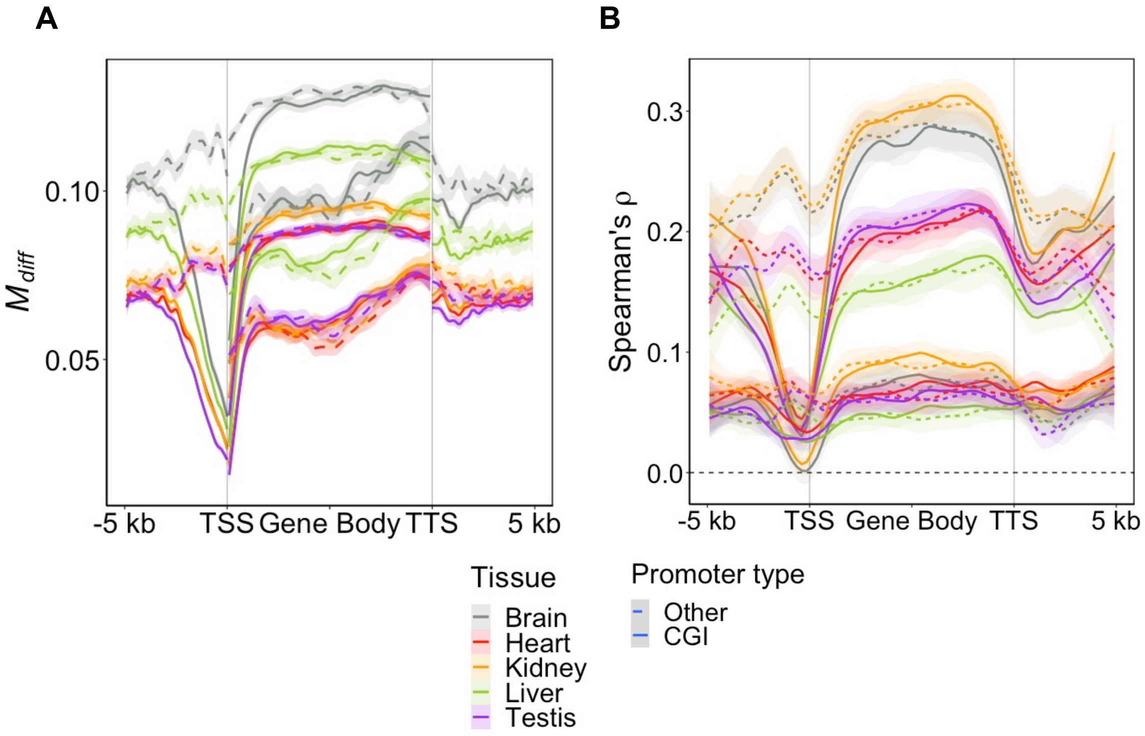

Figure S4: Gene profile of the proportion of CGIs. Vertical lines demarcate the TSS and TTS as well as the beginning of the 2kb promoter region.

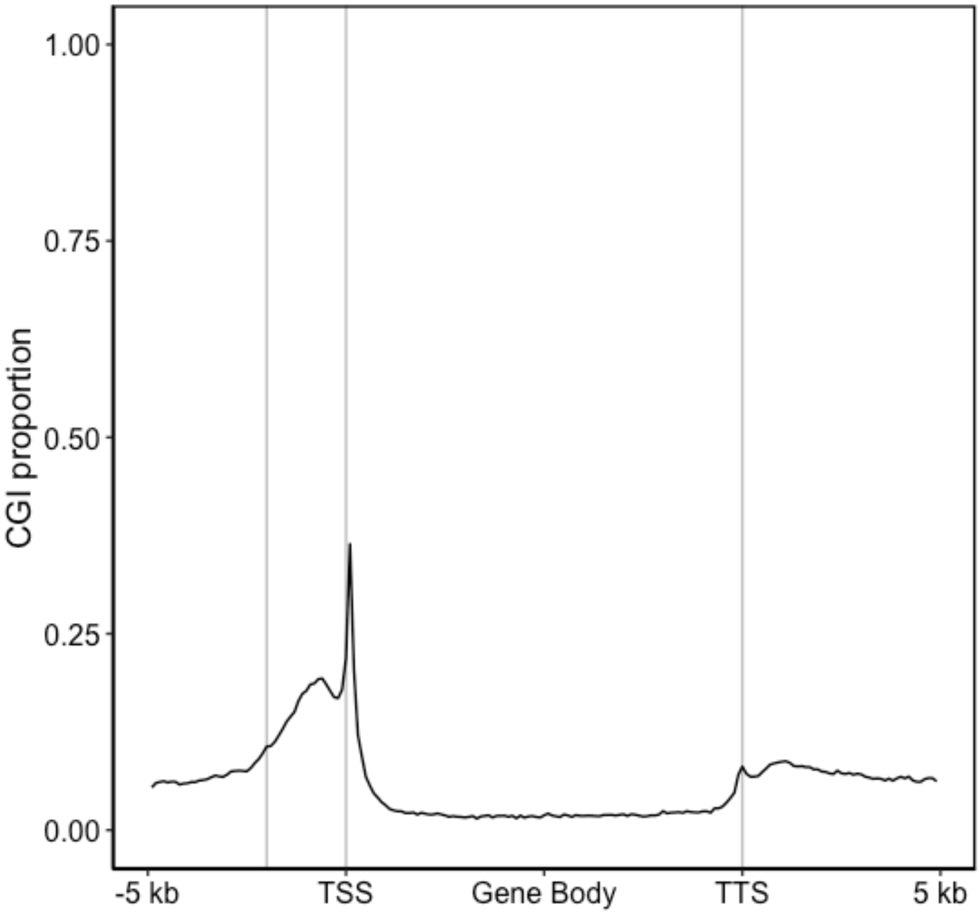

Figure S5: Patterns of genetic and epigenetic change at DE genes between HYB and both COL and PIE (misexpressed genes). (A) and (D) show DMR frequency, while (B) and (D) show non-CpG F_{ST} between COL and PIE. Testis and brain not included in (E) and (F) for visibility due to very few DE genes.

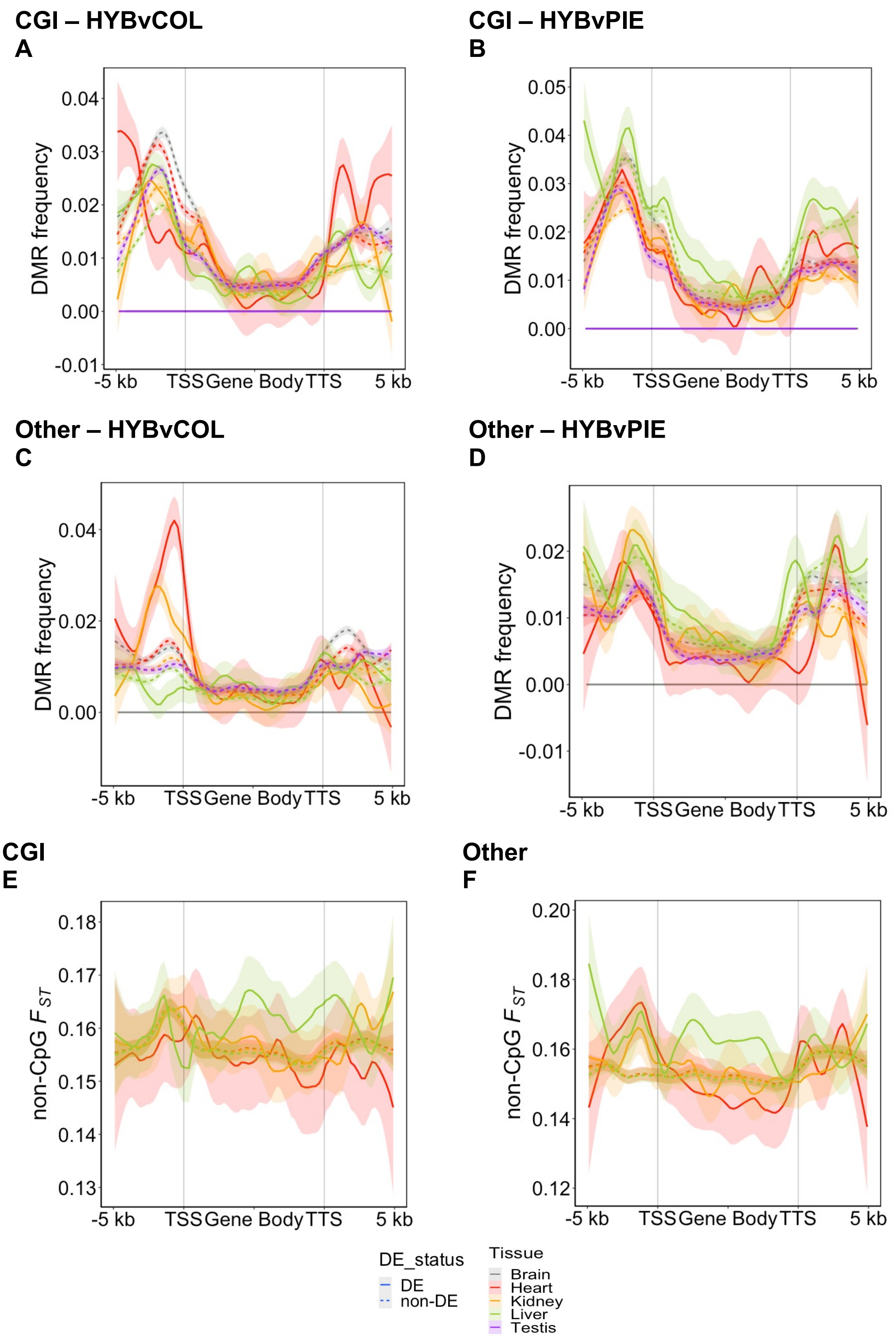

Table S1: Sample and sequencing information (see external file).

Table S2: Frequency of hypermethylation of tissue-specific DMRs. Here being hypermethylated means that a tsDMR has a higher methylation level in a specific tissue compared to the others. For example, a majority of testis tsDMR were hypermethylated for COL, PIE and HYB.

	Brain	Heart	Kidney	Liver	Testis
**COL**	0.61 ***	0.16 ***	0.13 ***	0.33 ***	0.77 ***
**PIE**	0.56	0.13 ***	0.12 ***	0.23	0.68 ***
**HYB**	0.43	0.19 ***	0.10 ***	0.17 ***	0.74 ***

Family-wise (0.1) adjusted p-value levels * < 0.05 ** < 0.005 *** < 0.0005

Table S3-6: tsDMR GO analyses (see external files).

Table S7: Classification system for the mechanism of DNA methylation divergence. Classification was based on significance or not in pairwise comparisons between the parental (PAR) species, between alleles in the hybrids (HYB) and between the same allele dependent on parental species background (COL: PARvHYB and PIE: PARvHYB).

**PAR: COLvPIE**	**HYB: COLvPIE**	**COL: PARvHYB**	**PIE: PARvHYB**	**Results**
**N**	**N**	**N**	**N**	Conserved
**N**	**N**	**S**	**N**	Conserved
**N**	**N**	**N**	**S**	Conserved
**N**	**N**	**S**	**S**	Conserved
**S**	**S**	**N**	**N**	Cis
**S**	**S**	**S**	**N**	Cis + Trans or Cis x Trans
**S**	**S**	**N**	**S**	Cis + Trans or Cis x Trans
**S**	**N**	**S**	**N**	Trans
**S**	**N**	**N**	**S**	Trans
**S**	**N**	**S**	**S**	Trans
**N**	**S**	**S**	**N**	Compensatory
**N**	**S**	**N**	**S**	Compensatory
**N**	**S**	**S**	**S**	Compensatory
**S**	**S**	**S**	**S**	Cis + Trans or Cis x Trans

S = Significant

N = Non-significant

Table S8: Classifications of fixed difference loci according to methylation patterns in parental species and hybrids. Most loci did not fit the stringent filtering criteria (see main text and Supplementary Methods) and were classified as ambiguous. A majority of loci passing the filtering criteria showed conserved methylation patterns between COL and PIE.

Chr. type	Tissue	Ambiguous	Conserved	Cis	Trans	Compensatory	Cis x Trans	Cis + Trans
A	Brain	30104	2916	2	16	4	0	0
A	Heart	27556	5247	20	32	20	6	0
A	Liver	27308	5377	30	36	21	7	1
A	Testis	25998	6714	30	29	41	2	0
Z	Brain	3823	309	1	1	2	0	0
Z	Heart	3496	602	6	2	1	0	0
Z	Liver	3449	631	6	6	2	0	1
Z	Testis	3206	858	12	10	4	0	0

Table S9: Number of differentially- and non-differentially expressed genes in different comparisons split by promoter type.

		COL v PIE		HYB v COL		HYB v PIE		Misexpressed	
**Tissue**	**Promoter type**	DE	Non-DE	DE	Non-DE	DE	Non-DE	DE	Non-DE
Brain	CGI	52	4946	1	4967	9	4959	1	4967
Heart	CGI	156	4842	240	4727	416	4551	133	4834
Kidney	CGI	308	4690	533	4435	1047	3921	340	4628
Liver	CGI	242	4756	549	4415	894	4070	342	4622
Testis	CGI	687	4311	24	4944	44	4924	1	4967
Brain	Other	29	3391	6	3393	5	3394	1	3398
Heart	Other	84	3336	162	3234	263	3133	95	3301
Kidney	Other	222	3198	332	3067	585	2814	200	3199
Liver	Other	178	3242	355	3043	536	2862	209	3189
Testis	Other	475	2945	30	3369	32	3367	0	3399

Table S10: Determinants in *cis* of overdominant and underdominant genes (misexpressed genes). Average DMR frequency differences and non-CpG F_{ST} (between parental species) in the 2k upstream promoter region and throughout the gene body were compared between DE and non-DE genes using paired *t*-tests. The table displays p-values of those tests. Brain and testis were excluded since they had too few DE genes.

**Tissue**	**Promoter type**	**P_{DMR} (HvP) freq.**	**GB_{DMR} (HvP) freq.**	**P_{DMR} (HvC) freq**	**GB_{DMR} (HvC) freq**	**P_{non-CpG} *Fst***	**GB_{non-CpG} *Fst***
Brain	CGI	NA	NA	NA	NA	NA	NA
Heart	CGI	1 (-)	1 (-)	0.053 (-)	0.002 (-) **	1	1 (-)
Kidney	CGI	1 (-)	1 (-)	1 (-)	0.028 *	1 (-)	1
Liver	CGI	1	0.77	1	1 (-)	1 (-)	0 ***
Testis	CGI	NA	NA	NA	NA	NA	NA
Brain	Other	NA	NA	NA	NA	NA	NA
Heart	Other	1	0.124 (-)	0.001 **	1	0.321	0.011 (-) *
Kidney	Other	0.104	1	0.087	1 (-)	1	1
Liver	Other	1	0.341	0.001 (-) *	0.04 *	1	0 ***
Testis	Other	NA	NA	NA	NA	NA	NA

HvP = Hybrids versus pied flycatchers
HvC = Hybrids versus collared flycatchers
(-) = Average value lower in DE genes
P = Promoter
GB = Gene body
Family-wise (0.1) adjusted p-value levels * < 0.05 ** < 0.005 *** < 0.0005
